# Dietary Interventions and Nutritional Factors in the Prevention of Pediatric Asthma

**DOI:** 10.3389/fped.2020.00480

**Published:** 2020-08-18

**Authors:** Irene Trambusti, Giulia Nuzzi, Giorgio Costagliola, Elvira Verduci, Enza D'Auria, Diego G. Peroni, Pasquale Comberiati

**Affiliations:** ^1^Section of Pediatrics, Department of Clinical and Experimental Medicine, University of Pisa, Pisa, Italy; ^2^Department of Health Sciences, University of Milan, Milan, Italy; ^3^Department of Pediatrics, Vittore Buzzi Children's Hospital, University of Milan, Milan, Italy

**Keywords:** asthma, breastfeeding, children, complementary foods, omega-3 long-chain polyunsaturated fatty acids (LCPUFAs), primary prevention, probiotics, Vitamin D

## Abstract

Asthma is the most frequent chronic disease in children, and its pathogenesis involves genetic, epigenetic, and environmental factors. The rapid rise in the prevalence of asthma registered over the last few decades has stressed the need to identify the environmental and modifiable factors associated with the development of the disease. In particular, there is increasing interest in the role of modifiable nutritional factors specific to both the prenatal and post-natal early life as, during this time, the immune system is particularly vulnerable to exogenous interferences. Several dietary factors, including maternal diet during pregnancy, the duration of breastfeeding, the use of special milk formulas, the timing of the introduction of complementary foods, and prenatal and early life supplementation with vitamins and probiotics/prebiotics, have been addressed as potential targets for the prevention of asthma. In this review, we outline recent findings on the potential role of prenatal and perinatal dietary and nutritional interventions for the primary prevention of pediatric asthma. Moreover, we addressed unmet needs and areas for future research in the prevention of childhood-onset asthma.

## Introduction

Asthma is the most frequent chronic disease in children and is associated with significant morbidity and potential impairment of lung function in adulthood (1). Both genetic, epigenetic, and environmental determinants are involved in the pathogenesis of asthma. Childhood-onset asthma, as opposed to adult-onset asthma, is typically featured by a history of atopy and related markers of type-2 allergic inflammation (2). From 1960, the prevalence of allergy and asthma has progressively increased worldwide, reaching a plateau in some developed countries (3), while continuing to rise in low-income and mid-income countries (4). As a result, over the last few decades, there has been increasing interest in the identification of risk factors for allergy and asthma (5, 6). Recent research has focused on tackling both prenatal and postnatal modifiable factors for the prevention of allergic diseases, as these factors can influence the immune system in a crucial phase of its development (7). A relationship between environmental tobacco smoke, air pollution, respiratory infections, and the development of asthma has been demonstrated and extensively reviewed elsewhere (8, 9). On the contrary, the influence of *in utero* and early-life dietary and nutritional drivers needs to be more fully elucidated. The role of several dietary factors, including maternal diet and vitamin status, composition of the microbiome, duration of breastfeeding, the use of hydrolyzed formulas and the introduction of complementary foods, has been investigated in recent clinical trials, to identify potential targets for the prevention of childhood-onset asthma (10).

This review focuses on the latest findings on the role of prenatal and perinatal dietary factors in the development of asthma, and whether the modulation of such factors could contribute to the primary prevention of childhood-onset asthma.

## Maternal Diet During Pregnancy

Given the central role of the first 1,000 days of life for the development of the immune system, numerous studies have investigated the role of intrauterine exposures in the pathogenesis of allergic diseases (11). Recent evidence supports the hypothesis that colonization by a healthy gut microbiome during early infancy can affect the immune system development and the predisposition to immune- mediated diseases later in life, including asthma (12, 13). It has been shown that the maternal diet during pregnancy could influence the composition of the gut microbiome and the immune system development of the neonate, and therefore potentially affect the predisposition to asthma and allergies in childhood (14, 15). There is conflicting evidence on the role of prenatal maternal intake of certain foods and nutrients and the development of asthma and allergies during childhood. In 2015 Beckhaus et al. (16) reported that maternal *in-utero* intake of vitamin D (VD), vitamin E, and zinc had a protective effect against early life wheezing in offspring, but not on childhood-onset asthma or other atopic conditions. One recently published study, assessing the impact of pre-pregnancy diet on the risk of allergic outcomes in children (17), highlighted that the consumption of specific foods during pregnancy, such as cooked green vegetables and eggs, may protect against pediatric wheezing and asthma, while higher maternal intake of meat may increase the risk of wheezing, allergic rhinitis, and atopic dermatitis in children. Further studies are needed to confirm these results and understand the mechanisms related to maternal nutrition and the development of asthma and allergic disorders. Understanding the connection between maternal diet during pregnancy and neonatal microbiome composition may help identify effective prevention strategies, such as providing pregnant women and women desiring pregnancy with nutrition recommendations, particularly regarding products that may influence the development of allergic diseases.

## Maternal Vitamins Intake During Pregnancy

The recent increase in the prevalence of asthma and allergic disorders in Westernized countries has closely paralleled a VD deficiency epidemic (18). Experimental evidence shows that VD contributes to the fetal-neonatal lung growth and modulates both innate and adaptive immune responses, inhibiting some pro-inflammatory responses associated with allergy and asthma (19). These findings have supported the hypothesis that maternal VD status may play a role in the development of pediatric asthma. Three observational studies found that the risk of asthma in school-age decreases as maternal VD levels increase (20–22), although a recent meta-analysis of observational studies showed that lower prenatal exposure to VD was associated with an increased risk of respiratory infections, but not with asthma and allergic rhinitis development (23). In an early randomized controlled trial (RCT), Goldring et al. (24) randomized 180 women at 27 weeks of gestation to receive either no VD, 800 IU/VD2 daily until delivery or single oral bolus of 200,000 IU/VD3, reporting no reduction in wheezing in offspring at 3 years of age. In the more recent Vitamin D Antenatal Asthma Reduction Trial (VDAART), 881 pregnant women with high atopy risk were randomized to receive either 4,400 IU VD3/daily (active group) or 400 IU VD3/daily (control group), during the 2nd and 3rd semesters of gestation (25). The offspring of the active group showed a clinically relevant, although not statistically significant, reduction (20% or greater) in the risk of asthma/recurrent wheezing by age 3 years, but not by age 6 years (25, 26). These results are in line with those reported by a similar 6-year follow-up trial, the COPSAC study (27, 28), in which pregnant women from an unselected cohort were randomized to receive either 2,400 IU or 400 IU VD3/daily during the 3rd trimester of gestation. These findings show that increasing VD supplementation during pregnancy may reduce the incidence of transient pre-school wheezing, but is not sufficient to prevent school-age asthma, which is usually an allergic-type of asthma (29) (Table 1). Further studies are needed to address the influence of VD maternal status on the development of asthma in children before high dose VD supplementation could be recommended during pregnancy.

**Table 1:** Randomized controlled trials on vitamin D supplementation for the prevention of childhood wheezing/asthma.

**Authors, Years**	**Population (N), characteristics**	**Time of exposure**	**Interventions**	**Outcomes**
Goldring et al. (24)	180 pregnant women	Prenatal	No VD vs. 800 IU VD2 daily from 27 weeks of gestation until delivery vs. single oral bolus of 200,000 IU VD3 at week 27 of gestation	Wheezing illnesses (assessed by validated questionnaire) in offspring at age 3 years
Litonjua et al. (25) Litonjua et al. (26)	876, pregnant women (18–39 years) high-risk cohort for asthma	Prenatal	4,400 IU VD3/d vs. 400 IU VD3/d starting at 10–18 weeks of gestation until delivery	Asthma or recurrent wheezing in offspring at age 3 years Asthma or recurrent wheezing in offspring at age 6 years
Chawes et al. (27) Brustad et al. (28)	623, pregnant women, unselected cohort	Prenatal	2,400 IU VD3/d vs. 400 IU VD3/d (control) starting at 24 weeks of gestation until delivery	Persistent wheeze and asthma in offspring at age 3 years Asthma in offspring at age 6 years
Grant et al. (30)	260, pregnant women and their infants	Pre and postnatal	Woman-Infant pairs receiving: Placebo-placebo vs. 1,000 IU - 400 IU VD3/d vs. 2,000 IU - 800 IU VD3/d from 27 weeks gestation to birth, and then to offspring for the first 6 months of life	Aeroallergen sensitization and healthcare visit for acute respiratory illness in offspring at age 18 months
Hibbs et al. (31)	300, black premature infants (born at 28–36 weeks' gestation)	Postnatal	400 IU VD3/d vs. diet-limited supplementation daily from birth to 6 months of life	Recurrent wheezing in offspring by 12 months' adjusted age

*IU, International Units; VD, vitamin D*.

Regarding other vitamins, Stone et al. (32) showed that higher levels of vitamin E, particularly in its alpha-tocopherol isoform, in postpartum maternal plasma concentration were associated with a decreased likelihood of asthma and wheezing over 2 years.

## Breastfeeding

Breastfeeding is the most relevant postnatal factor that supports microbial colonization and drives the immune system development of the newborn (33, 34). Compared with formula feeding, breastfeeding has been related to lower morbidity and mortality in infants and decreased incidence of allergic diseases, including asthma (35). Increasing evidence shows that breast milk plays a central role in the development of tolerogenic immune responses during the first years of life, due to its content in immunoglobulins, vitamin A and transforming growth-factors, which promote the gut mucosal barrier integrity and homeostasis (35, 36). Significantly, it has been observed that certain aeroallergens such as dust mite allergens, when found in breast milk, can increase the risk for developing food allergy, as they could disrupt intestinal immune homeostasis through their protease activity and prevent the induction of oral tolerance to food allergens (37). Regarding asthma, breast milk appears to have a protective, dose-dependent impact on respiratory health, especially for preschool wheezing, although the mechanisms of how breastfeeding reduces the risk of childhood wheezing/asthma is not fully elucidated (38). A systematic review by Dogaru et al. (39) showed that children who were breastfed longer had a lower risk of developing childhood wheezing/asthma. This finding was more significant at ages 0–2 years, and diminished over time, although it remained evident at school age (39).

Other systematic reviews and meta-analyses reported similar findings, suggesting that breastfeeding is protective against pre-school wheezing, which is commonly triggered by viral respiratory infections, whereas this protection tended to wane in older children when heterogeneous factors can influence respiratory morbidity (38, 40). Although there is a growing body of evidence on the role of breast milk in the development of immune tolerance during the first months of life, the impact of breastfeeding on asthma pathogenesis remains controversial.

## Hydrolyzed Formula Feeding

The role of partially and extensively hydrolyzed milk formulas (pHF and eHF, respectively) in asthma prevention is still controversial. Two early RCTs showed no significant difference between pHF and eHF in the prevention of allergic diseases in children, including asthma (41, 42). In a prospective, double- blind RCT, high-risk children that could not be breastfed were randomized to receive whey-based pHF or eHF, casein-based eHF, or standard cow's milk formula (CMF) for the first 4 months of life. Hydrolysate nutrition did not have any preventive effect either on asthma or early and late wheezing (43). After 10 years of follow-up, the authors observed no effects on the development of asthma (44), while, between 11 and 15 years of age, the prevalence of asthma was lower in the casein-based eHF group than in the CMF group. No significant effect was found in the whey-based eHF group on any manifestation, nor was there any effect on sensitization with any formula (45).

In a recent birth cohort study, infants received breast milk only, pHF with or without a hypoallergenic label, or non- hydrolyzed formula. The use of the pHF-with hypoallergenic label, compared to non-hydrolyzed formula, had no protective effect on the risk of asthma up to 2 years of age and was related to a higher risk of wheezing at 1 year in high-risk infants (46). A recent Cochrane review concluded that the use of hydrolyzed formula in the early days of infancy, compared to exclusive breastfeeding, showed no significant differences in terms of infant allergy prevention. In particular, the authors found no evidence to support the use of pHF compared to CMF to prevent allergic diseases among non- exclusively breastfed infants (47).

## Postnatal Vitamin D Intake

The hypothesis that VD status in childhood might influence the susceptibility to childhood asthma and allergy is supported by the evidence on the role of VD as a key modulator of lung growth and innate and adaptive anti-inflammatory immune responses (48–50). Experimental data have shown that low VD levels are associated with increased type 2-mediated responses, interleukin-10 production, and reduced T-regulatory cells (48). In addition, recent data have shown that early postnatal colonization of the airways by pathogenic bacteria, a risk factor for the development of asthma, is influenced by VD status (50). VD may also affect airway remodeling by direct inhibition of airway smooth muscle cell growth and contractility and fibroblast proliferation (51). Observational studies have shown that VD deficiency in early life is associated with the occurrence and persistence of childhood asthma (19, 52). In a high-risk Australian cohort, VD deficiency in early childhood was associated with a higher risk for persistent asthma at 10 years of age (53). VD deficiency in infancy was also associated with increased risk of early allergic sensitization and susceptibility to respiratory infections (53), which are known risk factors for both preschool and school-age asthma (54).

Interestingly, VD supplementation during pregnancy and infancy has been related to a reduced risk of sensitization to house dust mites at age 18 months (30).

The results of the D-Wheeze trial, in which 300 black infants born prematurely were randomized to receive either a sustained supplementation with 400 IU/day of VD (active group) or a diet-limited supplementation (control group) showed a 34% reduced risk for recurrent wheezing by 12 months in the intervention group (31). There is also evidence that VD supplementation can decrease susceptibility to respiratory viral infection in older children (55). Taken together, the results from *in-utero* (29) and post- natal VD supplementation trials support a role for VD in reducing susceptibility to preschool viral wheezing illnesses. However, there are insufficient data to address whether postnatal VD supplementation may help in the primary prevention of persistent school-age asthma (56), and proper intervention trials with long-term follow-up are needed (Table 1). Intervention trials assessing the combination of prenatal and postnatal VD supplementation would also be needed before VD supplementation can be recommended for the primary prevention of pediatric wheezing and asthma.

## Probiotics and Prebiotics

There is mounting evidence showing the relationship between the composition of the early-life gut microbiome and the risk of asthma in children (57, 58), which has promoted studies on the modulation of the gut microbiome as a means of preventing asthma. However, RCTs of probiotic and prebiotic supplementation for the prevention of pediatric asthma have shown mixed efficacy outcomes (Table 2). An early systematic review and meta-analysis showed no protective role of oral probiotic supplementation during pregnancy or early life on the development of childhood wheeze and asthma (68). A study by Peldan et al. (61) in high-atopy risk children showed that prevalence of asthma at 5 and 10 years of age was similar in the group receiving oral probiotics mixture for the first 6 months of life (also including maternal supplementation starting at week 36 of gestation) and the placebo group. A recent meta-analysis of RCTs concluded that probiotic supplementation during pregnancy or early life did not influence the incidence of wheeze or asthma in infants, but seemed to reduce wheezing incidence among the subgroup of infants with atopic disease (69).

**Table 2:** Randomized controlled trials on prebiotics/probiotics and LCPUFA supplementation for the prevention of childhood wheezing/asthma.

	**Authors, Years**	**Population (N), characteristics**	**Time of exposure**	**Interventions**	**Outcomes**
Prebiotics Probiotics	Arslanoglu et al. (59)	134, high-risk infants	Postnatal	8 g/L scGOS/lcFOS vs. placebo during the first 6 months of life	Allergic manifestations and infections during the first 2 years of life
	Niele et al. (60)	113, preterm infants	Postnatal	prebiotic mixture of 80% scGOS/lcFOS and 20% pAOS vs. placebo between days 3 and 30 of life	Allergic manifestations and infections during the first year of life
	Peldan et al. (61)	1,223, pregnant women and their offspring	Pre and postnatal	mixed of LGG, L. rhamnosus LC705, Bifidobacterium breve Bb99 and Propionibacterium freudenreichii ssp. shermanii JS vs. placebo (prebiotics) twice daily from 36 weeks of gestation until delivery and in infants daily for the first 6 months of life	Allergic manifestations during the first 10 years of life
LCPUFA	Dunstan et al. (62)	98, pregnant atopic women	Prenatal	3.7 g n-3 PUFAs vs. placebo daily from 20 weeks of gestation until delivery	Allergy, including asthma at 1 year of age
	Bisgaard et al. (63)	736, pregnant women	Prenatal	2.4 g n-3 LCPUFA (fish oil) vs. placebo (olive oil) daily from 24 weeks of gestation until delivery	Persistent asthma and wheezing at 5 years of age
	Olsen et al. (64)	533, pregnant women	Prenatal	2.7 g n-3 PUFAs vs. capsules with olive oil vs. no oil capsules from 30 weeks of gestation until delivery	Asthma at 16 years of age
	D'Vaz et al. (65)	420, high-risk infants	Postnatal	280 mg docosahexaenoic acid and 110 mg eicosapentaenoic acid vs. placebo (olive oil) from birth to age 6 months	Eczema, food allergy and asthma at 1 year of age
	Mihrshahi et al. (66)	376, high-risk infants	Postnatal	tuna fish oil and omega-3-rich margarine and cooking oils vs. placebo (polyunsaturated margarine and cooking oils) from 6 months of life (or at the start of formula feeding)	Allergic sensitization and asthma/wheezing at 18 months of age
	Marks et al. (67)	516, high-risk children	Postnatal	House dust mite avoidance (mattress cover) vs. placebo Dietary fatty acid modification (see (66)) vs. placebo	Asthma, allergic sensitization and eczema at 5 years of age

*Cfu, colony forming units; lcFOS, longchain fructo oligosaccharides; LCPUFA, omega-3 long-chain polyunsaturated fatty acids; scGOS, short-chain galacto-oligosaccharides; pAOS, pectin-derived acidic oligosaccharides*.

In a 2-year follow-up RCT involving 132 infants at risk of atopy, infants that were fed with a formula containing a mixture of prebiotic oligosaccharides reported a lower incidence for recurrent wheezing compared to the placebo group (59). In a 1-year follow-up RCT involving 113 preterm infants, supplementation of non-human neutral and acidic oligosaccharides during the neonatal period did not reduce the incidence of allergies, bronchial hyper-reactivity, and respiratory infections (60). In 2013, a Cochrane review reported no significant effect of oral prebiotics for the prevention of childhood- onset asthma (70). A more recent meta-analysis of RCTs concluded that the role of prebiotics in the prevention of allergies is still uncertain (71).

Taken together, the evidence on the effects of oral probiotics and prebiotics for the prevention of pediatric asthma is so controversial that no definitive recommendation can be made. Differences in the probiotic strain specificity, the population treated, the timing of administration, and the duration of the intervention all contribute to the heterogeneity of the meta-analysis and of RCT outcomes.

## Omega-3 Long-Chain Polyunsaturated Fatty Acids

The supplementation with omega-3 long-chain polyunsaturated fatty acids (LCPUFA) during pregnancy and early life, through the administration of fish oil, has been proposed for the prevention of allergic sensitization and atopic disease, including asthma (72, 73). LCPUFA influence the membrane structure and function, potentially modulating the function of the cells involved in the immune and inflammatory response (74). Most of the studies on maternal fish oil supplementation during pregnancy showed a reduced risk of allergic sensitization to both foods and aeroallergens in children (62, 75–78). In some of these studies, also a lower incidence of eczema was reported (77, 78), while other authors reported no differences in the incidence of allergic diseases (76, 79). Two meta-analyses reported a beneficial effect of maternal supplementation with fish oil on the reduction of allergic sensitization and eczema (80), and on the sensitization to egg and peanut (81), respectively. However, a recent Cochrane review concluded that there is “limited evidence” to support that supplementation with LCPUFA during pregnancy and lactation could reduce the incidence of allergies in children (82). Regarding the prevention of wheeze/asthma, the literature data available show mixed efficacy results for the supplementation of LCPUFA during pregnancy (62–64) (Table 2). Indeed, a recent meta-analysis concluded that LCPUFA supplementation during pregnancy is not associated with a significant protective effect on wheeze/asthma in offspring (81).

Conflicting results have been found in studies investigating fish oil supplementation in infants and children for the prevention of allergic sensitization and asthma (65–67) (Table 2). A meta-analysis of RCTs found no evidence of a protective role of LCPUFA supplementation in infants and children in the prevention of asthma (83). Overall, the available studies show methodological heterogeneity and risk for suboptimal adherence bias. Therefore, further trials are needed to clarify the role of LCPUFA supplementation during pregnancy and early life in the prevention of pediatric asthma.

## Timing of Complementary Feeding

Recent advances in the field of allergy prevention showed that early 2000s recommendation to delay the introduction of solid allergenic foods to the infant's diet is not an effective approach to reduce the risk of allergic sensitization and atopic diseases in children (84–88). More recently, high-quality clinical trials showed that the early introduction of some food allergens, such as peanut and egg, is associated with a reduced risk of food allergy to those foods (84). There is conflicting evidence regarding early complementary feeding and the prevention of pediatric asthma, with some observational studies reporting that the early introduction of some solid foods (before 1 year of age), such as oat, fruit, vegetables, and fish, was associated with a reduced prevalence of wheezing and asthma in childhood (89–91), while others reporting no such association (92).

The timing of the introduction of fish is of particular interest to the purpose of primary prevention of asthma, given its high content in LCPUFA. Despite the heterogeneity in the methods of analysis and outcome measures, the early introduction of fish has been associated, in many observational studies, with a reduced risk of allergic sensitization (93, 94). However, the protective effect of early introduction of fish on the development of infant wheezing and asthma seems more controversial, with some observational studies reporting such association (93, 95–98), while others did not confirm this protective effect (90, 99). Indeed, two recent meta-analyses conclude that introducing fish before the age of 9 months is associated with low-to-very low evidence of reduced allergic sensitization and rhinitis (94), but there is limited evidence that early introduction of fish could reduce the risk of developing wheezing and asthma in childhood (100).

## Conclusion

The significant increase in the prevalence of asthma and allergic diseases registered in recent years has promoted research on the identification of modifiable risk factors for the prevention of such disorders. It is well-acknowledged that respiratory health is determined by a complex interaction between genetic factors and environmental drivers that occur during prenatal and early postnatal life, including dietary factors. However, it remains difficult to define the contribution of specific dietary supplements and nutritional food sources to the risk of developing pediatric asthma, due to the heterogeneous pathogenesis of this disease and the limitations of the currently available evidence, in terms of study design, type and duration of interventions and outcomes measures.

Further research is needed to accurately identify dietary and nutritional modifiable risk factors for asthma and to address whether the modulation of such factors, either alone or in combination, could contribute to the primary prevention strategies of pediatric asthma.

## Author Contributions

DP, GN, IT, GC, and PC made substantial contributions to conception, design, and acquisition of data. GC, IT, GN, and PC drafted the initial manuscript. DP, EV, ED'A and PC critically reviewed it for important intellectual content. All authors approved the final version of the manuscript. All authors contributed to the article and approved the submitted version.

## Conflict of Interest

The authors declare that the research was conducted in the absence of any commercial or financial relationships that could be construed as a potential conflict of interest.
